# Development of an inducible excision system of a visual marker *Ipomoea batatas Myb* gene from the genome of transgenic cells

**DOI:** 10.5511/plantbiotechnology.23.0309a

**Published:** 2023-06-25

**Authors:** Yuka Sato, Mayu Fukuda, Peter Nkachukwu Chukwurah, Tomoko Igawa

**Affiliations:** 1 Graduate School of Horticulture, Chiba University, 648 Matsudo, Matsudo-shi, Chiba 271-8510, Japan; 2 Plant Molecular Science Center, Chiba University, 1-33 Yayoi, Inage-ku, Chiba-shi, Chiba 263-8522, Japan

**Keywords:** *Cre*-*loxP* recombination system, heat-inducible promoter, *IbMyb*, visible marker

## Abstract

In the plant genetic transformation process, single selection by a chemical-resistant marker gene occasionally allows the proliferation of non-transgenic cells, escaping selection pressure. The additional use of a visual marker gene is effective for accurate selection. For instance, R2R3-MYB genes are used for regulating anthocyanin biosynthesis; however, constitutive *Myb* expression in transgenic plants is not always desirable and may cause developmental abnormalities due to excess anthocyanin accumulation. To overcome the remaining problems in the use of *Myb* as a visible marker, we developed T-DNA. *Ipomoea batatas* *Myb* (*IbMyb*) and *Cre* expression cassettes were inserted between two *loxP* sequences, and the *hygromycin phosphotransferase* (*HPT*) and *green fluorescent protein* (*GFP*) expression cassettes were located outside of the *loxP*-*IbMyb*-*Cre*-*loxP* region. In the developed system, *IbMyb* and *Cre* were excised from the genomes of transgenic cells using heat-inducible *Cre*-*loxP* recombination. Upon heat treatment in a general incubator, green shoots emerged from purple tobacco transgenic calli that were pigmented with *IbMyb* expression. The excision of *IbMyb* from the genome of green shoots was confirmed using polymerase chain reaction (PCR) and sequencing. GFP expression was observed in the roots of the obtained green transgenic plants. We report that the system developed here operated successfully in tobacco, showing the potential to provide an easier and cheaper visual selection of transgenic cells in the genetic transformation process.

Plant transformation through artificial gene transfer is considered a critical technology in plant science and the development of breeding programs. In the transformation procedure, the gene of interest is introduced with a selection marker gene to distinguish transgenic cells following the culture process. Chemical selection markers such as herbicide- (bialaphos, sulfonylurea, etc.) and antibiotic- (hygromycin, kanamycin, gentamycin, etc.) resistance genes are routinely used, whereby the proliferating cells that survive against these chemical reagents are successively isolated and subcultured as putative transgenic cells. However, the proliferation of non-transgenic cells escaping selection pressure occasionally occurs. In such cases, distinguishing between transgenic and non-transgenic cells based only on their proliferation phenotype is challenging. Some studies reported the presence of chimeric regenerated plants for the transgene (Christou and Ford 1995; Domínguez et al. 2004; Hinchee et al. 1988; Rakosy-Tican et al. 2007), whereby the cause behind chimera production is attributed to the phenomenon of early transient expression of the marker gene and the multicellular origin of the regenerated tissue (Faize et al. 2010). To avoid chimera production, a *green fluorescent protein* (*GFP*) gene was introduced to label the transgenic cells (Rakosy-Tican et al. 2007). Visual selection of *GFP*-expressing cells requires additional equipment such as a stereo microscope with an excitation light system. The equipment must be placed on a clean bench during aseptic manipulation, as prolonged exposure of the excitation light to cultures may harm the cells and lead to photobleaching (Dixit et al. 2006). Additionally, clear GFP signals are difficult to observe because of autofluorescence in tissues containing chloroplasts (Dixit et al. 2006). Therefore, the development of an alternative visual selection system would be helpful for plant transformation studies.

Anthocyanins are natural pigments that are widely found in plants, whereby they synthesize and accumulate in the colors red, blue, or purple. To date, some R2R3-MYB transcription factors have been reported to regulate anthocyanin biosynthesis. *Myb* genes have been used as visible markers in sweet potato, wheat, rubber tree, grape, tobacco, apple, strawberry, and tomato (Gao et al. 2011; Huang et al. 2021; Jin et al. 2016; Kandel et al. 2016; Kim et al. 2010; Kortstee et al. 2011; Lim et al. 2012), allowing for selection by the naked eye. However, the colored appearance of transgenic plants due to constitutive *Myb* expression is not always desired. Additionally, anthocyanin over-accumulation sometimes causes abnormalities in morphology and sterility (Mano et al. 2007).

To overcome the remaining problems with *Myb* utilization as a visible selection marker gene, we developed an improved system using the *Ipomoea batatas* *Myb* (*IbMyb*) gene (Mano et al. 2007) and *Cre*-*loxP* recombination system (Zhang et al. 2003) (Figure 1). The fragment of *IbMyb* cassette (35S^P^-*IbMyb*-NosT) was cloned into the pCR™8/GW/TOPO™ vector (Thermo Fisher Scientific, Inc., M.A., USA) and then transferred to the pMDC99*_rescue* vector (Nagahara et al. 2015) through recombination mediated by LR Clonase™ (Thermo Fisher Scientific K. K., Tokyo, Japan), producing the pMDC99*_rescue*:*IbMyb* vector. The fragment of the *IbMyb* and *Cre* (HSP^P^-*Cre*-NosT) cassettes including the *loxP* sequence at both ends were amplified using polymerase chain reaction (PCR), then inserted into the Eco53kI site in the pMDC99 vector through In-Fusion® (Takara Bio Inc., Shiga, Japan), producing the pMDC99:*IbMyb*/*Cre* vector. The *GFP* expression cassette (Ubi^P^-*GFP*-NosT) cloned into pCR™8/GW/TOPO™ (pCR8:*GFP*) was transferred into pMDC99:*IbMyb*/*Cre* through an LR reaction. Finally, the obtained pMDC:*IbMyb*/*Cre*/*GFP* vector comprised four gene expression cassettes: *IbMyb*, *Cre*, *hygromycin phosphotransferase* (*HPT*), and *GFP*. The constructed T-DNA region is shown in Figure 1.

**Figure 1:**
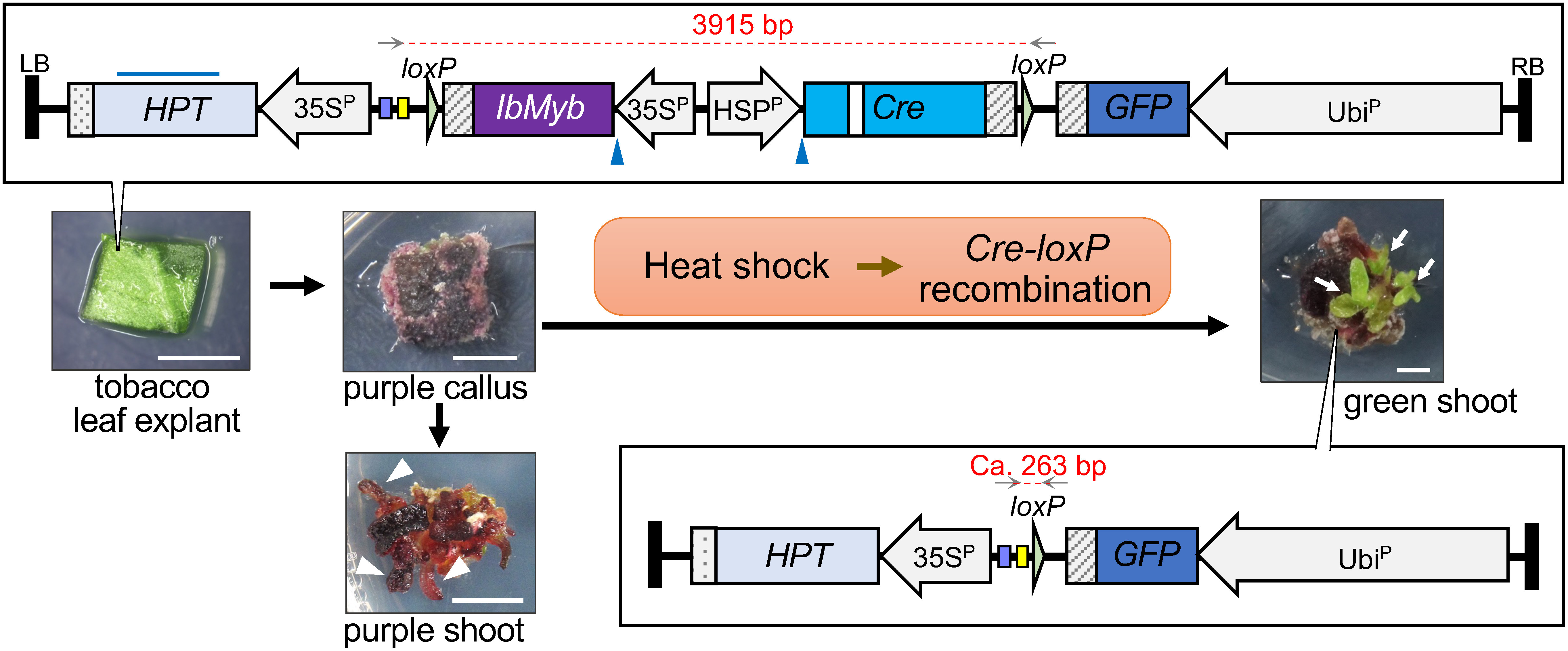
Figure 1. Schematic diagram of the removable visible marker system developed in this study. The flowchart of transformation and heat treatment procedures are drawn. The T-DNA region (top of the picture) from the binary vector pMDC:*IbMyb*/*Cre*/*GFP* (15,876 bp) was introduced into tobacco leaf explants through *Agrobacterium* transformation. The *Cre* coding region contains an intron (internal white box). The induced callus was colored purple due to the constitutive expression of *IbMyb* driven by the cauliflower mosaic virus 35S promoter (35S^P^). The adventitious shoots that emerged from the purple calli were also colored purple (arrowhead). By heat treatment, *Cre* under the control of the heat shock promoter (HSP^P^) was expressed, leading to *Cre-loxP* recombination and the excision of *IbMyb* and *Cre* cassettes from the genome of transgenic cells (bottom of the picture). As a result, green shoots emerged from the purple calli (white arrows). Gray arrows above the T-DNA region indicate corresponding locations of the PCR primers used to evaluate excision by *Cre-loxP* recombination. Each amplicon size before/after excision is indicated above the dashed red line. The blue bar above the *HPT* and the two positions marked with blue arrowheads indicate the probe region and the HindIII recognition sites used for Southern blot analysis. Light purple and yellow boxes flanking the left *loxP* represent the lac promoter and M13 reverse sequences, respectively, which are unique in the T-DNA region. The oblique lined and dotted boxes are the Nos terminator and CaMV polyA signal, respectively. RB: right border, LB: left border. Bars=5 mm.

*Cre* expression is controlled by the soybean heat shock protein promoter (HSP^P^) (Prändl and Schöffl 1996). The *Cre* coding region contains an intron sequence to prevent unexpected excision in *E. coli* and *Agrobacterium*. *HPT* was introduced for chemical selection and *GFP* was used as a model gene of interest. *IbMyb* and *Cre* cassettes were located between the *loxP* sequences; hence, these cassettes were expected to be excised by the synthesized CRE after heat treatment. The obtained vector was named pMDC:*IbMyb*/*Cre*/*GFP* (15,876 bp) and the entire T-DNA region was confirmed using sequencing. Primers used in this study are listed in Supplementary Table S1.

Mature tobacco leaves (*Nicotiana tabacum* ‘Petit Havana SR1’) (Maliga et al. 1973) were used for plant transformation. *Agrobacterium tumefaciens* strain EHA105 containing pMDC:*IbMyb*/*Cre*/*GFP* was grown in Luria Broth (LB) liquid medium containing 20 mg l^−1^ rifampicin, 30 mg l^−1^ chloramphenicol, and 50 mg l^−1^ kanamycin at 28°C overnight in a shaking incubator (120 rpm). *Agrobacterium* solution was diluted to an optical density measured at 600 nm (OD_600_) of 0.1 with Murashige-Skoog (MS) liquid medium containing 3% sucrose and 100 µM acetosyringone. Mature tobacco leaves were cut into 5-mm squares and soaked in *Agrobacterium* solution for 15 min. The leaf explants were then cultured on an MS medium containing 3% sucrose and 0.8% agarose for 3 days in the dark. Subsequently, the explants were moved to an MS selection/differentiation medium containing 0.1 mg l^−1^ 1-naphthaleneacetic acid (NAA), 1.0 mg l^−1^ 6-benzylaminopurine (BA), 40 mg l^−1^ hygromycin B, 20 mg l^−1^ meropenem (Sumitomo Dainippon Pharma Co., Ltd., Tokyo, Japan), 3% sucrose, and 0.8% agar. After four weeks of culture, purple calli and shoots originated from the leaf explants. Purple shoots were detached and grown on MS medium containing 40 mg l^−1^ hygromycin B, 20 mg l^−1^ meropenem, 3% sucrose, and 0.8% agarose for plant regeneration. As a control plant, a transgenic line Myb^OE^ overexpressing *IbMyb* and *HPT*, but not containing a *Cre-loxP* system, was produced with the pCam*-35S_IbMyb* vector (provided by Dr. Ootani and Mr. Nakayachi). No morphological abnormalities were found in the Myb^OE^ plants, except for pigmentation in whole tissues (Supplementary Figure S1).

To confirm the integration of the T-DNA region in transgenic plants, Southern blot analysis was performed on two independent transgenic lines (Myb/Cre #1 and #4). Genomic DNA was isolated from 100 mg of fresh young leaves using the cetyltrimethylammonium bromide (CTAB) method. The plasmid (pMDC:*IbMyb*/*Cre*/*GFP*) and genomic DNA from Myb^OE^ were used as experimental controls. *HPT* probes were synthesized using the PCR DIG Probe Synthesis Kit (Roche Diagnostics K. K., Tokyo, Japan) using the primers shown in Supplementary Table S1. The genomic DNA and plasmids were digested with the restriction enzyme, HindIII. The following procedure was performed according to the manufacturer’s instructions. The results showed that both lines had two copies of the transgenes in the genome (Supplementary Figure S2).

For the analysis, the callus was induced again from the leaf explants of each transgenic line using an MS selection/differentiation medium. The purple callus was cut into 5 mm blocks, and seven blocks of calli were inoculated onto the MS selection/differentiation medium in a plate (diameter=90 mm, depth=20 mm) (Supplementary Figure S3A). Heat treatment was performed at 37°C or 42°C for 6 h, and the calli were returned to 25°C. The heat treatment was performed one or two times, with a 24-h interval at 25°C between each treatment. Three biological replicates were carried out for each transgenic callus line. Continuous incubation at 25°C was performed as the mock treatment (Supplementary Figure S3B). Thirty-one days after the first heat treatment, the number of green shoots that emerged from the purple callus block was counted to represent the *IbMyb* excision using heat-inducible CRE (Supplementary Figure S3C). The total number of green shoots produced in each treatment is listed in Table 1. In Myb^OE^, green shoot production was not significantly different under any of the heat treatment conditions. Statistical analysis for each heat treatment condition was performed for Myb/Cre #1 and Myb/Cre #4 in comparison with Myb^OE^, and the *p*-values were calculated using Welch’s *t*-test (unequal variances *t*-test). Green shoot production frequency was increased significantly by all heat treatments in Myb/Cre #4 at *p*<0.05 level (Table 1). In contrast, Myb/Cre #1 showed a significant increase in green shoot production when calli were treated at 37°C or twice at 42°C (*p*<0.05). The observed difference between the two transgenic lines could be due to the loci of the transgenes influencing the CRE-mediated recombination. Nevertheless, the two heat treatments at 42°C induced significantly higher green shoot production in both Myb/Cre transgenic lines (*p*<0.01) (Table 1). Green shoots that emerged from the purple calli (Figure 1 and Supplementary Figure S3C) were detached and grown on MS medium containing 40 mg l^−1^ hygromycin B, 20 mg l^−1^ meropenem, 3% sucrose, and 0.8% agar to promote root induction. The GFP fluorescence signal was clearly observed in the roots of the regenerated plants from the green shoots, showing a normal phenotype without anthocyanin accumulation (Figure 2A).

**Table 1:** Table 1. Number of green shoots that emerged from transgenic purple callus after heat treatment.

Heat treatment conditions (x; performed times)	Total number of the emerged green shoots				
	Myb^OE^	Myb/Cre #1		Myb/Cre #4	
25°C (continuous)	3 (0, 3, 0)	8	(3, 3, 2)	0	(0, 0, 0)
37°C x 1	0 (0, 0, 0)	6*	(2, 1, 3)	6*	(0, 3, 3)
37°C x 2	1 (1, 0, 0)	13*	(2, 8, 3)	16**	(7, 4, 5)
42°C x 1	1 (1, 0, 0)	6	(1, 4, 1)	24**	(6, 1, 17)
42°C x 2	0 (0, 0, 0)	21**	(8, 5, 8)	19**	(6, 13, 0)

Three biological replicates were carried out for each treatment group. Numbers in parentheses indicate the number of green shoots obtained from 7 calli blocks in each replicate. Asterisks indicate significant differences relative to Myb^OE^ detected using Welch’s *t*-test (* *p*<0.05, ** *p*<0.01).

**Figure 2:**
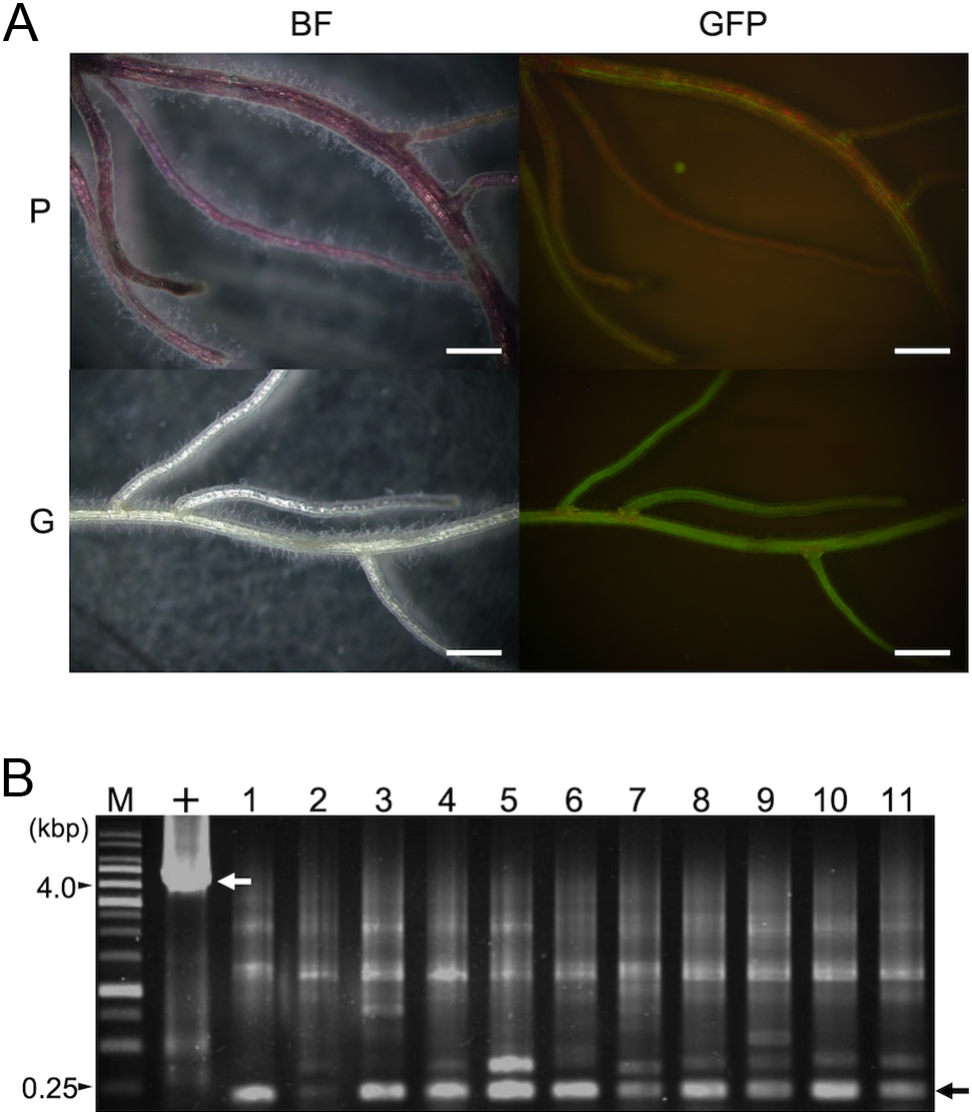
Figure 2. Confirmation of *IbMyb* excision and *GFP* expression in the green plant obtained after heat treatment. (A) GFP signals observed in the roots. P: purple-colored Myb/Cre #4 transgenic plant that has not experienced heat treatment; G: green Myb/Cre #4 transgenic plant obtained after heat treatment; BF: bright field image; GFP: GFP fluorescence image. Constitutive *IbMyb* expression impeded fluorescence capture to some extent (upper right panel). The stereomicroscopic images were captured with a DP-72 digital camera using Cellsense software (Olympus Co. Tokyo, Japan). Bars=1 mm. (B) Genomic PCR analysis to confirm *IbMyb* and *Cre* excision from the genome of Myb/Cre #4 green shoots (No. 1–11). The amplicons indicated with a black arrow were detected in all samples. The sequencing result of the corresponding band (No. 8) proved the excision (Supplementary Figure S4), whereas the upper bands were non-specific amplicons (data not shown). The amplicon marked with a white arrow was obtained with pMDC:*IbMyb*/*Cre*/*GFP* DNA (+), indicating a non-excised size of the fragment. M: 1 kbp DNA ladder marker (VIOLAMO-AS ONE, Osaka, Japan).

To confirm *IbMyb* excision from the genome of green shoots, genomic PCR with primers that annealed to the outside regions of the *loxP* sequences (Figure 1, Supplementary Table S1) was performed for 11 green shoots derived from the Myb/Cre #4 transgenic callus line. As a result, the smaller size of the band than the expected size (263 bp) was detected in all green shoot lines (Figure 2B). The sequencing of the purified band showed that both *loxP* sequences, in addition to the *IbMyb* and *Cre* expression cassettes, were removed, and 43 bases were inserted, reflecting recombination in the transgenic genome occurred (Supplementary Figure S4). Another genomic PCR and sequencing on a different green shoot showed a different excision pattern that a single *loxP*, *IbMyb*, and *Cre* were removed from the genome, and 19 bases were inserted (Supplementary Figure S4B).

In this study, we developed a binary vector loaded with schemes for the visual selection of *IbMyb* and its excision and verified the operation using tobacco transformants. Consequently, the colored transgenic cells were easily identified with the naked eye, and the *IbMyb* gene was eliminated from the genome using heat treatment in a general incubator. Almost all heat treatments induced green shoot production compared to the mock treatment, and the two heat treatments at 42°C showed a significant effect in both Myb/Cre transgenic lines (Table 1). However, the number of green shoots produced in each experiment showed high variability. This could be because of the experimental environment that cannot perform rapid heat transition in cell cultures using a general incubator. Both the Myb/Cre #1 and #4 lines had two copies of transgenes in the genome at different loci (Supplementary Figure S2) and showed different effects on green shoot production (Table 1). Therefore, the frequency of complete *IbMyb* excision may have been affected by the transgene loci. Future evaluation of the relationship between the number of integrated genes, loci, and excision frequency is essential for improving the accuracy of controlling this excision system.

While heat treatment is an easier manipulation for *Cre* induction at an arbitrary desired time, induction by chemical reagents such as dexamethasone would be applicable as a future improvement. Moreover, the application of other excision systems mediated by *piggyBac* transposase would be valuable because it enables the complete removal of the transgene at the target site (Nishizawa-Yokoi et al. 2015). Visual selection of transgenic cells with the naked eye without any specific equipment would facilitate the genetic transformation procedure. In this study, the expression of a single coding gene resulted in cell pigmentation. Recently, the *RUBY*, an artificial open reading frame that produces three enzymes required for betalain production, was developed as a non-invasive visible marker system (He et al. 2020). Although the *RUBY* region is longer than *IbMyb*, it may apply to an excision marker system as one of the alternative versions in the future. Further improvements of the system developed in this study can facilitate the process of plant transformation at a lower cost.

## Abbreviations

*HPT*: *hygromycin phosphotransferase*
HSP^P^: soybean heat shock protein promoter
*IbMyb*: *Ipomoea batatas Myb*

## Description of Supplementary Files

Supplementary Table S1. Primers used in this study.

Supplementary Figure S1. Myb^OE^ transgenic tobacco plant.

Supplementary Figure S2. Southern blot analysis of the transgenic plant lines used in this study.

Supplementary Figure S3. Calli and shoots produced in the evaluation of heat treatment conditions.

Supplementary Figure S4. Sequencing of the genomic PCR products from green shoots.
