# Supplementary material for: Development of an inducible excision system of a visual marker Ipomoea batatas Myb gene from the genome of transgenic cells
Source: Plant Biotechnol (Tokyo). 2023 Jun 25;40(2):175–9. doi: 10.5511/plantbiotechnology.23.0309a (PMC10797523; doi:10.5511/plantbiotechnology.23.0309a)
Supplement: Supplementary Data [file plantbiotechnology-40-2-23.0309a-s001.pdf]

Supplementary Files

Sato et al.

Supplementary Table S1. Primers used in this study

Primer name	Sequence (5'-3')	Applications
NosT_antisense	GATCTAGTAACATAGATGACACCGCGCG	Cloning of *IbMyb* cassette
Sal1_35Spro	GGTGGAGCACGACACACTTGTCTAC	Cloning of *IbMyb* cassette
NosT_Asc_infu	AGATCCCCCGGCGCGCCGATCTAGTAACATAGATGACACCGCGCG	Cloning of *GFP* cassette
Asc_intUbiP_infu	CAGGGTAATGGCGCGCCCTGCAGTGCAGCGTGACCCGGT	Cloning of *GFP* cassette
Cre_excisecheck_F	CAACGCAATTAATGTGAGTTAGCTCACT	Confirmation of excision
Cre_excisecheck_R	ACATGATGGCATATGCAGCATCTATTC	Confirmation of excision
exci.check_R	CCTTAATTAACTAGTTCTAGAGCGGCCG	Confirmation of excision
Eco53del_loxP_For	GATTACGAATTCGAGGCTCTGTTTCTCTCACCACAGC	Cloning of *IbMyb/Cre* cassettes
loxP_Rev_Eco53	GCCACCGCGGTGGAGCGGCTCTTGGTGCCATG	Cloning of *IbMyb/Cre* cassettes
Myb For	ATGGTTATTTCATCTGTA	Sequencing
UbiP(592-609)	CGCCGTCGACGAGTCTAA	Sequencing
UbiP(1191-1208)	GAATCCTGGGATGGCTCT	Sequencing
UbiP(1743-1760)	GATGTGGGTTTTACTGAT	Sequencing
pCam35S_seq.Rev	CGCAAGACCGGCAACAGG	Sequencing
IbMyb_seqF1	GGCCTCGACCTCGGAGATT	Sequencing
IbMyb_seqR1	GGGGAATTAGATGCCATTTT	Sequencing
HSpro_seqF1	CTCACTCTTTAAATACGATGTT	Sequencing
Cre_seqF1	GCGGCGGATCCGAAAAGA	Sequencing
Cre_seqF2	CAGTGCCCGTGTCGGAGC	Sequencing
Cre_seqR1	GGTGCTAACCAGCGTTTTCG	Sequencing
MYB_check_F	AAGGTTCATGGTCCGAAGAAGAAGAC	Sequencing
MYB_check_R	ACTCCAACAGATTTGTCCTTCCTCATT	Sequencing
HPT_Fw	GTGTCACGTTGCAAGACCTG	Probe preparation
HPT_Rv	CGAGTACTTCTACACAGCCATCG	Probe preparation

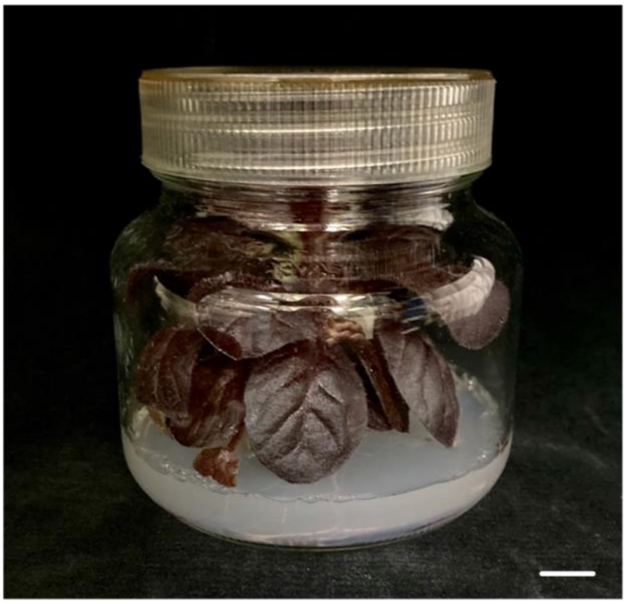

Supplementary Figure S1. Myb^{OE} transgenic tobacco plant.

Constitutive expression of *IbMyb* under the control of the cauliflower mosaic virus 35S promoter was sufficient for purple coloration of the plant tissue. In the case of *N. tabacum*, significant morphological abnormalities were not observed in this study. Bar = 1 cm.

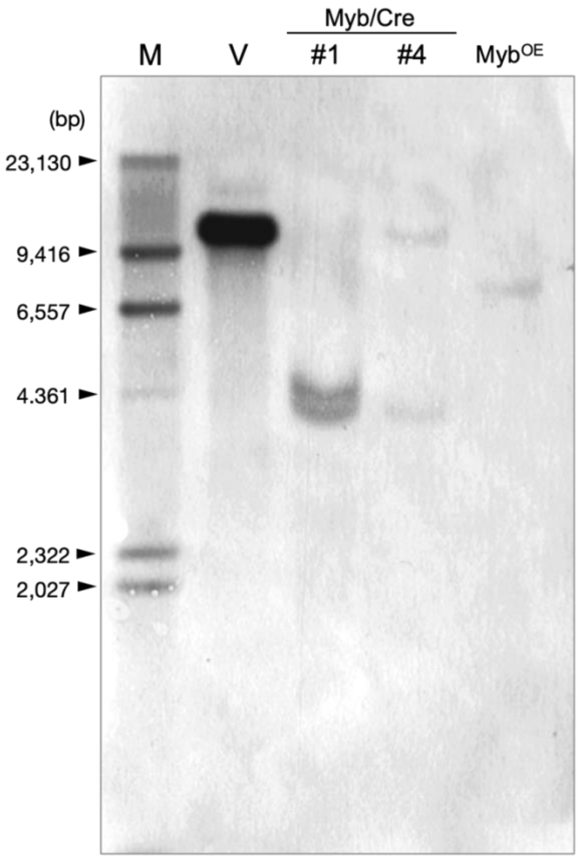

Supplementary Figure S2. Southern blot analysis of the transgenic plant lines used in this study. M: DNA molecular weight marker II, digoxigenin (DIG)-labeled (Roche Ltd., Basel, Switzerland); V: pMDC:*IbMyb*/*Cre*/*GFP* vector as the experimental positive control. Myb/Cre #1 and #4: independent transgenic lines produced using the pMDC:*IbMyb*/*Cre*/*GFP* vector. Myb^{OE}: transgenic line produced using the pCam-*35S_IbMyb* vector. The isolated genomic DNA was digested with HindIII and the *HPT* region was used as a probe.

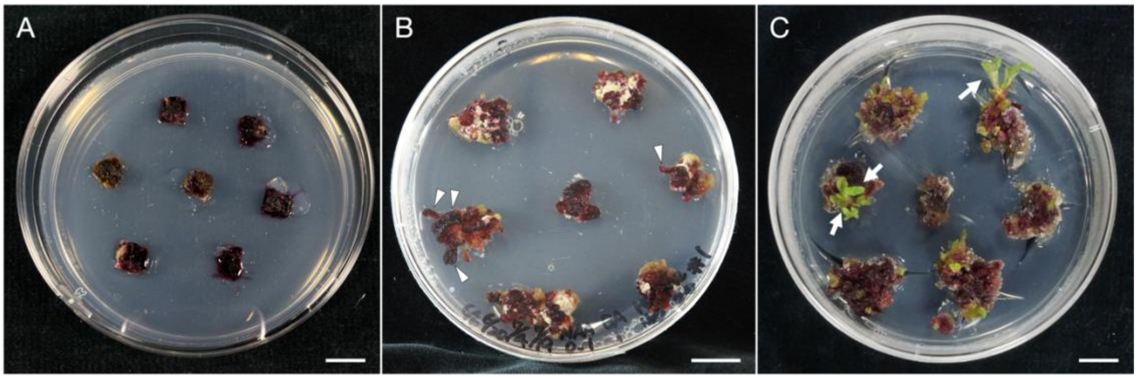

Supplementary Figure S3. Calli and shoots produced in the evaluation of heat treatment conditions.

The calli were cultured on MS selection/differentiation medium throughout the experiment. (A) Purple calli blocks before heat treatment. (B, C) The proliferated calli and the shoots produced on the purple calli 31 days after the first heat treatment. (B) Purple shoots (white arrowheads) were produced in the mock treatment (Myb/Cre #1 transgenic line). (C) Green shoots (white arrows) produced after two times of heat treatment at 37°C (Myb/Cre #4 transgenic line). Bars = 10 mm.

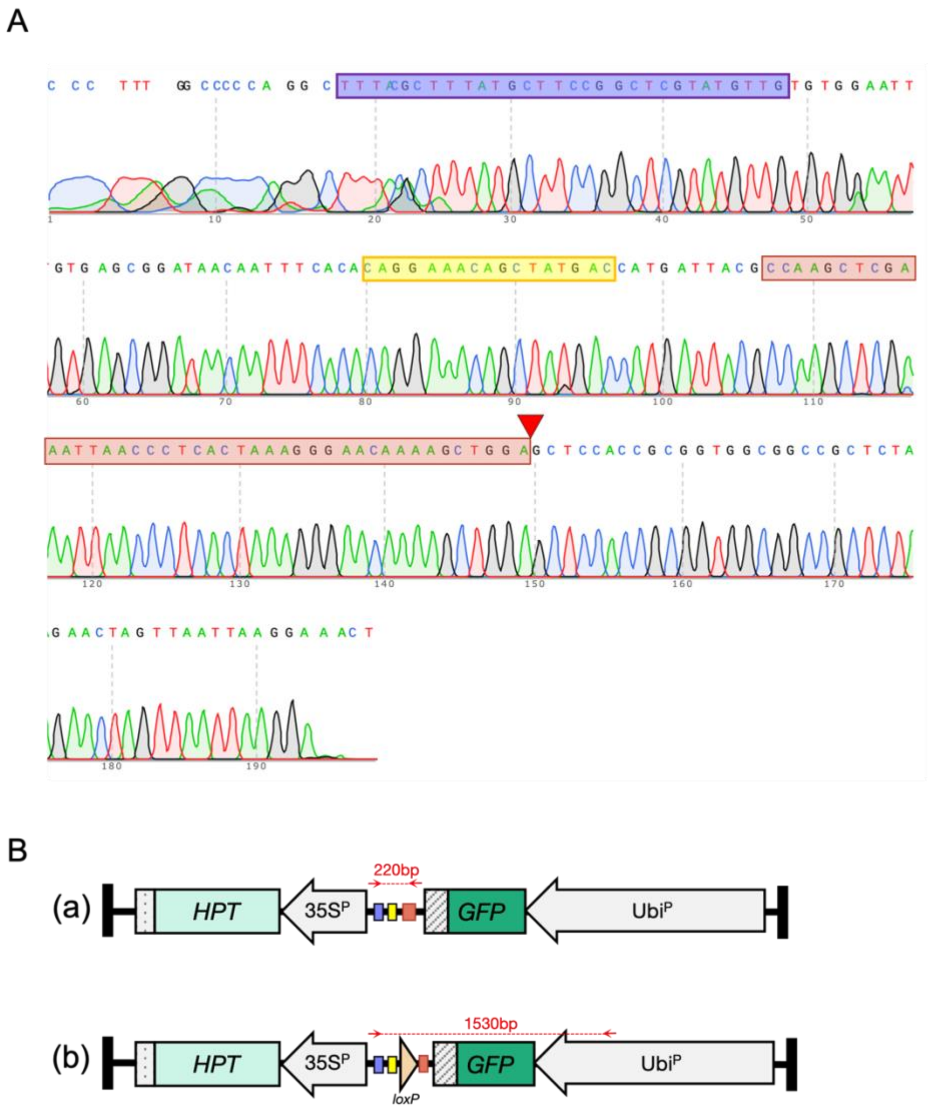

Supplementary Figure S4. Sequencing of the genomic PCR products from green shoots.

(A) Nucleotide sequencing waveform of the PCR product obtained with primers Cre_excisecheck_F and exci.check_R (Supplementary Table S1) using genomic DNA from a Myb/Cre #4 green shoot. The result from sample No. 8 (Figure 2B) was shown. The lac promoter and M13 reverse nucleotide sequences are enclosed with light purple and yellow boxes, respectively. The nucleotide sequence enclosed with a pink box represents the inserted bases. The red triangle indicates the junction after the excision. (B) Excision patterns detected in the different Myb/Cre #4 green shoots. (a) Schematic drawing of the result indicated in (A). In this excision pattern, the region of both *loxP* sequences, *IbMyb*, and *Cre* expression cassettes was removed, and 43 bases were inserted. (b) Another excision pattern was detected by PCR product sequencing using primers Cre_excisecheck_F and Cre_excisecheck_R (Supplementary Table S1). The region of a single *loxP* sequence, *IbMyb*, and *Cre* expression cassettes were removed, and 19 bases were inserted. The light purple, yellow and pink boxes represent lac promoter, M13 reverse, and inserted nucleotide sequences, respectively. Red arrows indicate corresponding locations of the PCR primers. The actual obtained size of each amplicon was indicated above the red dashed red line (bp).
